# A study of shape optimization on the metallic nanoparticles for thin-film solar cells

**DOI:** 10.1186/1556-276X-8-447

**Published:** 2013-10-29

**Authors:** Shiwei Zhou, Xiaodong Huang, Qing Li, Yi Min Xie

**Affiliations:** 1 Centre for Innovative Structures and Materials, School of Civil, Environmental and Chemical Engineering, RMIT University, GPO Box 2476, Melbourne 3001, Australia; 2 School of Aerospace, Mechanical and Mechatronic Engineering, The University of Sydney, Sydney, NSW 2006, Australia

**Keywords:** Thin-film solar cell, Nanoparticle, Shape optimization, 88.40.jm; 78.67.Bf

## Abstract

The shape of metallic nanoparticles used to enhance the performance of thin-film solar cells is described by Gielis' superformula and optimized by an evolutionary algorithm. As a result, we have found a lens-like nanoparticle capable of improving the short circuit current density to 19.93 mA/cm^2^. Compared with a two-scale nanospherical configuration recently reported to synthesize the merits of large and small spheres into a single structure, the optimized nanoparticle enables the solar cell to achieve a further 7.75% improvement in the current density and is much more fabrication friendly due to its simple shape and tolerance to geometrical distortions.

## Background

For some years, the high costs of silicon materials and fabrication make photovoltaics less competitive with electricity generation from fossil fuels even though it has potential to meet the soaring energy demands nowadays. Recently, many advanced light trapping techniques such as dye sensitization [1], plasmonic nanostructures [2-8], and nanodent plasmonic substrates [9-11] allow sunlight to be well absorbed within a very thin active absorber layer (just a few hundred nanometers). Therefore, the consumption of the absorber material was considerably reduced [2,3,7,12,13]. Remarkable outcomes have been achieved by depositing metallic nanoparticles into the dielectric layer between the Si layer and metallic back surface, by which the light path is optically prolonged as the sunlight can be scattered into the active layer at larger angles and induces extraordinarily strong local field intensity in the vicinity of metallic nanoparticles [2-5,8]. Such a phenomenon caused by the interaction of light with the nanostructures is termed as plasmon. Not all probable factors associated with thin-film solar cells such as the optical properties of constituting materials and environmental stimuli [6,14] have been thoroughly considered in the optimization, and this paper is confined to the influence of size and shape of nanoparticles only. In addition to various randomly shaped structures [3,4], the effects of some primitive geometries such as cylinder, cone, sphere, and hemisphere on light trapping have been investigated extensively [3,4,8,15,16]. It was reported that the cylindrical and hemispherical particles have better performance than spherical particles [4]. The investigation reveals that the Ag nanorod deposited on a SiO_2_ substrate has the strongest ability to enhance the resonance energy transfer rate when its cross section is a circle [17], which in turn proved the importance of the shape on the surface plasmon. Furthermore, a complicated two-level hierarchical nanostructure consisting of evenly distributed small spheres on the surface of a large sphere was found to have the virtues of both large and small spheres and therefore benefit the current enhancement considerably [5,18].

Nevertheless, complex shapes have not been explored for their plasmonic properties so far mainly due to the difficulties in 3-D modeling and optimization. As a class of unified mathematical expression, Gielis' superformula demonstrates its simplicity and generality of formulating a wide variety of 3-D geometries ranging from common shapes like sphere, cube, octahedron, and cylinder to highly complex structures via changing a small number of parameters [19]. A significant advantage of using Gielis' superformula for shape optimization is to facilitate the integration with evolutionary algorithm [20], enabling to search for such parameters by which the optimal structure can be constructed. In view of its recent success in investigating the geometry for a soft porous system (i.e., an adaptive structure undergoing large deformation [21] and plasmonic nanowires [22] in 2-D), it is prospective to explore this superformula in 3-D by seeking for the optimal nanoparticle for thin-film solar cells.

## Methods

**Figure 1:**
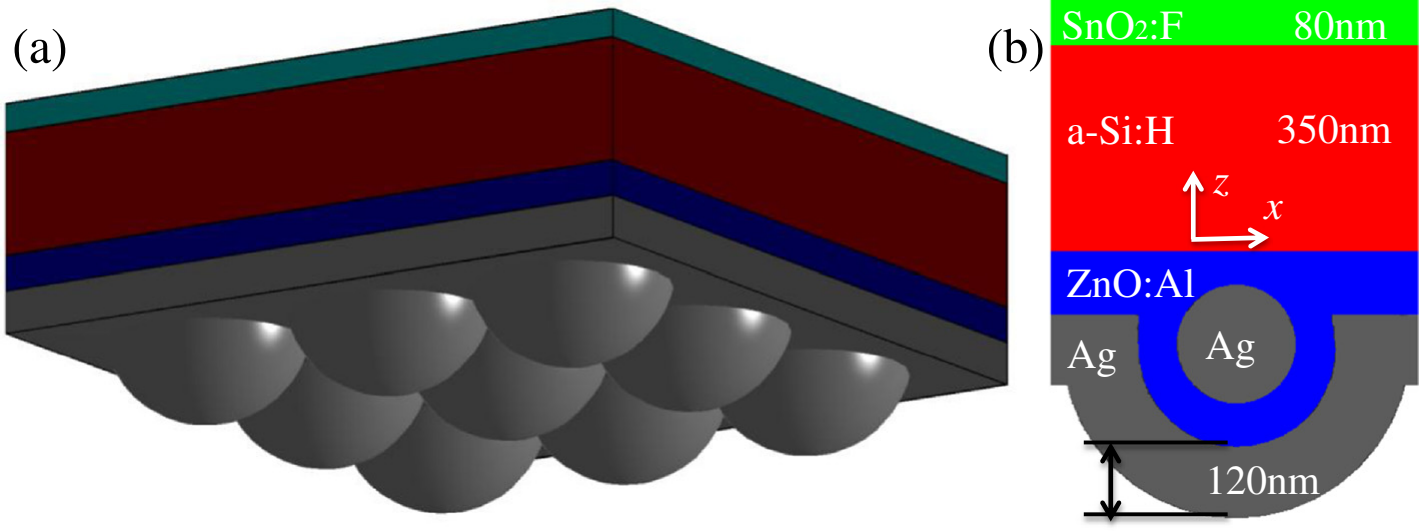
**Schematic and cross section of a thin-film solar cell. (a)** A 3-D schematic of a nanoparticle-enhanced thin-film solar cell. **(b)** Cross section of such a solar cell on the vertical symmetry plane.

As shown in Figure 1, the structure of a thin-film solar cell studied in this paper consists of four ordered layers: an 80-nm SnO_2_:F transparent conductive oxide layer on the front, followed by a 350-nm hydrogenated amorphous silicon (a-Si:H) active layer, subsequently a layer of Al-doped ZnO (ZnO:Al) thin film into which the Ag nanoparticles are periodically deposited, and finally a 120-nm Ag back reflector. The idea of such a layered configuration comes from the attempts in two-scale nanospheres, which is desirable for thin-film solar cells as the merits of large sphere (strong resonant intensity of field) and small spheres (large scattering angles) coexist [5,18,23].

Since the nanoparticles are periodically deposited and the incident light is normal to the front surface in this study, it is sufficient to restrain the modeling region to a representative volume element (RVE) with periodic boundary conditions applied to its bilateral surfaces parallel with the *z* axis. The open boundaries on the input and output sides are truncated by perfectly matched layers, whose distances to the top and bottom of the solar cell are 200 and 100 nm, respectively. In the RVE, the solar source is placed on a plane whose distance to the SnO_2_:F layer is 100 nm. The electromagnetic field within the RVE is governed by Maxwell's equations and solved by the finite difference time domain (FDTD) algorithm [24]. In the present study, we utilize the FDTD program from Lumerical [25] as it has been used by many researchers in this area.

In a spherical coordinate, the position of any surface point can be calculated as *x* = *r*(*θ*) cos(*θ*) *r*(*φ*) cos(*φ*), *y* = *r*(θ) sin(*θ*) *r*(*φ*) cos(*φ*), and *z* = *r*(*φ*) sin(*φ*), where the ranges of azimuthal and polar angles are –*π* ≤ *θ* ≤ *π* and –*π*/2 ≤ *φ* ≤ *π*/2, respectively*.* The radial function of Gielis shape is defined by

(1) $$r\left(\theta \right)={\left({\left|\text{cos}\left(\mathrm{m\theta}/4\right)/a\right|}^{n_{2}}+{\left|\text{sin}\left(\mathrm{m\theta}/4\right)/b\right|}^{n_{3}}\right)}^{-1/n_{1}}.$$

**Figure 2:**
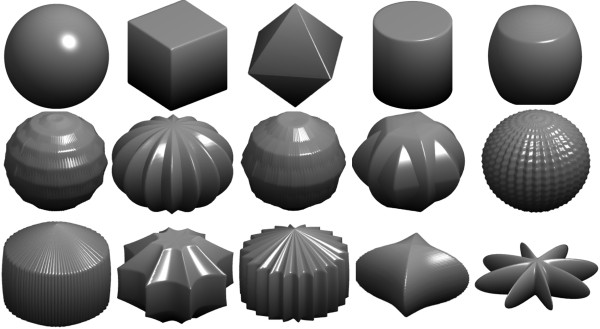
A variety of complex shapes in terms of Gielis' superformula.

The dimension of the 3-D object is determined by scaling factors *a* and *b*, while its shape is controlled by shape coefficients *n*_1_, *n*_2_, and *n*_3_. The number of rotational symmetries is governed by parameter *m*, which indicates the number of vertices in the *x*-*y* cross section. To increase the shape variety, *r*(*φ*) and *r*(*θ*) are different, determined by two sets of parameters (*m*, *n*_1_, *n*_2_, *n*_3_) and (*q*, *n*_4_, *n*_5_, *n*_6_), respectively. For the nanostructure considered in the thin-film solar cell, its shape is generally square symmetric in the plane normal to the incident wave (e.g., *x*-*y* plane); thus, *m* and *q* must be even numbers in the design. To decrease the design parameters, *n*_2_ = *n*_3_ and *n*_5_ = *n*_6_ can be imposed without influencing the diversity of Gielis shapes. Similarly, we let *a* = *b* = 1 and used a size weighting factor *s* to account for the volume and coverage of nanoparticles in the optimization. Following the aforementioned prerequisites, Figure 2 illustrates a class of interesting structures generated through variations on such a single mathematical equation. Some of the complicated 3-D pattern features are unlikely to be fabricated in nanoscale by conventional photolithography. However, it was reported very recently that the resolution limit of fabrication has been broken via a combination of electron beam lithography, photolithography, and resist spray coating. Structures with high aspect ratio have effectively been produced in the submicron scale via this new technique [26]. Therefore, the structures characterized with elaborated features in Figure 2 can be fabricated in nanoscale upon further research.

Since a larger short circuit current density *J*_sc_ indicates a higher open circuit voltage and higher conversion efficiency for solar cells, it is selected as the cost function to be maximized in the optimization. If each electron–hole pair contributes to the photocurrent, namely 100% internal quantum efficiency, *J*_sc_ is commonly defined as

(2) $$J_{\mathrm{sc}}=e\int \lambda /\left(\mathrm{hc}\right)\mathrm{QE}\left(\lambda \right)I_{\mathrm{AM}1.5}\left(\lambda \right)\mathrm{d\lambda},$$

where *e* denotes the charge on an electron, *h* the Plank constant, *λ* the wavelength, and *c* the speed of light in vacuum. The quantum efficiency QE(*λ*) = *P*_abs_(*λ*)/*P*_in_(*λ*) is the ratio of the power of the absorbed light *P*_abs_(*λ*) to that of the incident light *P*_in_(*λ*) within the active film. *I*_AM1.5_ stands for the relevant part of the solar spectral irradiance. Once Maxwell's system is solved, the *J*_sc_, electric intensity, absorption, as well as other relevant factors can be computed to any predefined level of accuracy.

Based on the above description, the optimization becomes a problem when searching for a stationary point in the hyperspace defined by seven design variables (*s*, *m*, *n*_1_, *n*_2_, *q*, *n*_4_, *n*_5_), so an extreme value of the cost function is achieved. Now that the number of design variables is relatively small, we adopt the evolutional algorithm [20] for the optimization. Such a non-gradient method is rather simple but fairly efficient and is especially suitable for optical optimization in which the gradient of the cost function with respect to the design variables is often too sensitive to be controlled and stabilized numerically in terms of our previous studies [22,23,27-29]. The evolutionary algorithm starts from a set of parent vectors randomly selected in the given parametric space. For each parent vector, the fitness, namely short circuit current density *J*_sc_, is calculated. Then, an offspring is introduced by a mutation rule defined as the summation of the weighted difference between a pair of parent vectors and the third one. The offspring is required to mend by a cross-over process for improving the diversity. In this step, the weighting factor and cross-over probability are set to be 0.5 and 1, respectively, for the following examples. The mutation and cross-over processes are repeated until the best performance is obtained. Importantly, predefined constraints like the bounds of design variables are incorporated into the optimization algorithm to avoid missing feasible solutions and generating unmanufacturable structures.

## Results and discussion

**Figure 3:**
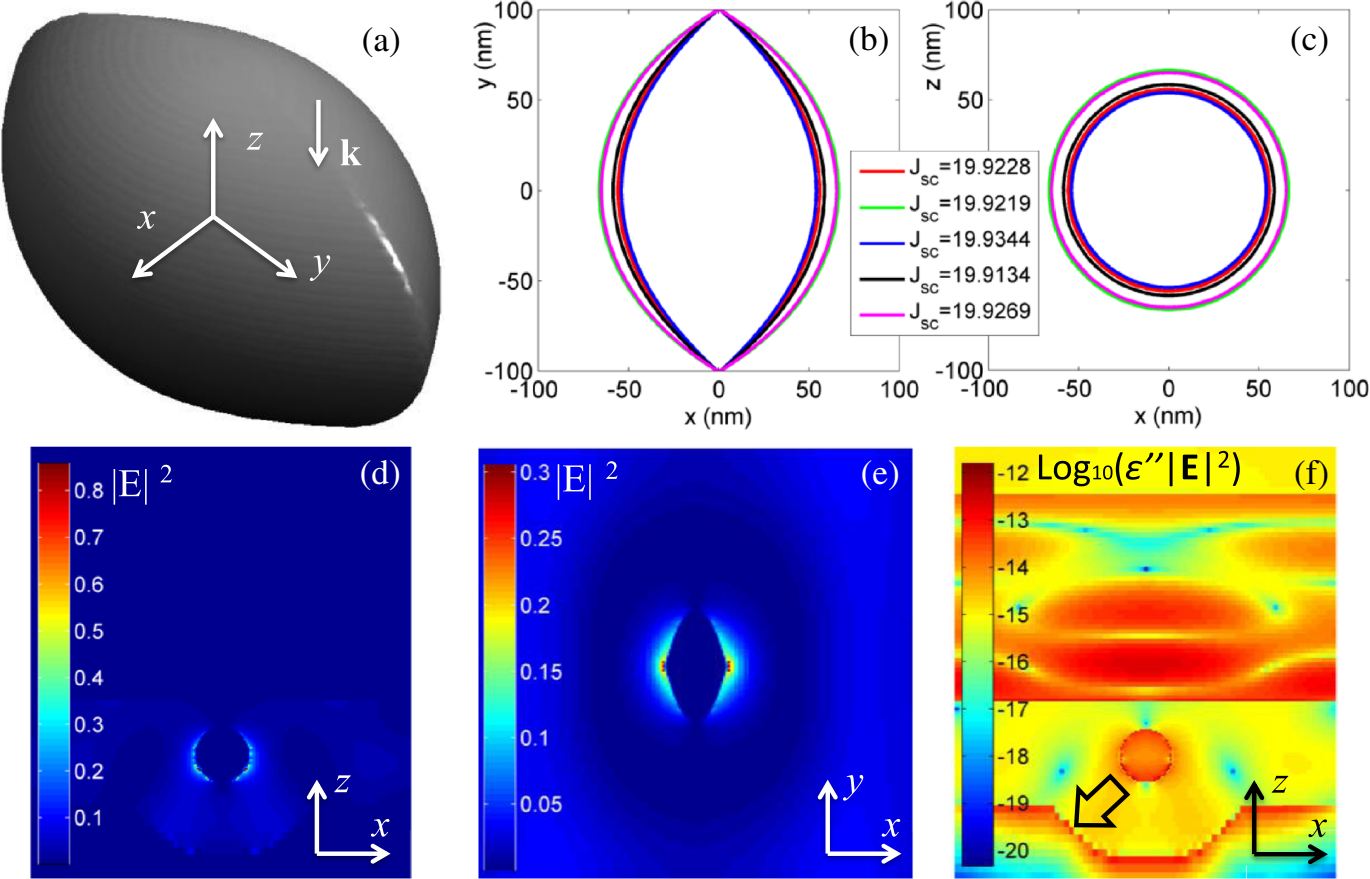
**Optimization process results. (a)** 3-D view of the optimized lens-like nanostructure. **(b**, **c)** The attained *J*_sc_ and cross-sectional profiles of the lens-like particles on *x*-*y* and *x*-z planes. **(d**, **e)** The electric intensity on the cross section of the lens-like nanoparticle on *x*-z and *x*-*y* planes. **(f)** Contour of the logarithmic scale of the absorbed energy per unit volume on the *x*-z plane.

As the thin-film solar cell utilizing two-scale nanospheres [5] has been employed as a benchmark, all the solar cells studied thereafter have the same parameters (e.g., periodicity of nanostructure) for a rational comparison. Some intermediate structures, together with the design parameters and generated current density, are listed in Table 1, which clearly illustrate the enhancement of *J*_sc_ and the capability of scanning complex structures in the optimization. It should be noted that not all structures in Figure 2 are examined, as the evolutionary algorithm skips the ones that generate lower *J*_sc_ if a better structure has been identified in previous iteration steps. Finally, the optimization process yields a lens-like structure (Figure 3a) that makes the solar cell exhibit *J*_sc_ = 19.93 mA/cm^2^, improving the *J*_sc_ = 18.50 mA/cm^2^ of the abovementioned benchmarking solar cell by 7.73%. Several similar structures obtained in the final steps of the optimization are presented in Figure 3b,c, respectively, showing their contours in the horizontal and vertical symmetric planes. Interestingly, they generate a very close outcome of *J*_sc_ ranging from 19.91 to 19.93 mA/cm^2^, even though their shape difference is notable. Such a robust performance is of critical importance to mass production as certain fabrication errors, usually unavoidable in nanoscale, can be tolerated by such a reliable structure. Moreover, compared to the two-scale nanosphere [5], such a lens-like structure is relatively easier to be fabricated due to its simpler geometry.

**Table 1:** Intermediate nanoparticles in the optimization

***J***_**sc**_	***m***	***n***_**1**_	***n***_**2**_	***q***	***m***_**1**_	***m***_**2**_	***s***	**Nanoparticle**
17.9778	142	6,917.3889	188.7937	6	246.5756	337.3163	71.1604	
18.6231	158	19,964.3647	711.4337	10	15,278.7838	1,812.3877	81.6840	
19.1814	72	1,774.9	74.6116	12	1,315.1222	473.4534	49.0618	
19.4887	10	1,113.2549	196.8687	116	1,268.8916	3.2545	43.1573	

The Gielis parameters for this optimal lens-like structure are *m* = 2, *n*_1_ = 767.6272, *n*_2_ = 1,379.6088, *q* = 152, *n*_4_ = 18,071.6197, and *n*_5_ = 255.3518, which result in 4.86% nanoparticle coverage for the thin-film solar cell when the periodicity of nanoparticles is 646.58 nm and the weighting factor is *s* = 45.08. As the coverage is much smaller than the widely accepted optimal coverage (10%) [5] with randomly shaped nanoparticles, the fabrication cost is likely to be significantly reduced because less silver material is consumed. On the other hand, the volume ratio of this structure to the benchmarking two-scale sphere is around 14.76%, indicating that the increase of *J*_sc_ is attributed to the shape rather than the volume because larger nanoparticles generally induce stronger surface plasmon and therefore a larger *J*_sc_. Additional evidence supporting this claim is that *J*_sc_ is not directly related to the areas enclosed by the contours as demonstrated in Figure 3b,c.

A glimpse of the near-field optical contour helps to explain the enhancement of short circuit currency density. Figure 3d,e illustrates the near-field electric intensity (|**E**|^2^) on vertical and horizontal symmetry planes, respectively, at the time when |**E**|^2^ reaches the maximum on the horizontal symmetry plane (the distribution of the **E** field is recorded by a time monitor in the simulation). It is seen that the near-field intensity is evidently strengthened in the bottom part (Figure 3d) and the bilateral vertices (Figure 3e) around the nanoparticle. The solar cell can make use of such a strong near-field enhancement to increase the optical absorption in the active layer, thereby amplifying *J*_sc_. Interestingly, similar shapes to the cross-sectional geometry of these lens-like structures have been reported elsewhere by Macías et al. recently when they attempted to search for the 2-D shapes with maximal scattering geometry [22]. Such a correlation signifies the crucial role played by this structure in inducing strong surface plasmon.

The logarithmic scale of the absorbed power per unit volume ∫ *ωϵ*″|**E**|^2^*dV*/2 (*ω* is the angular frequency and *ϵ*'' the imaginary part of permittivity) in different layers of the solar cell on the vertical symmetry plane at the frequency where the maximal absorption is attained (*λ* = 596 nm) is plotted in Figure 3f. It is noted that the enhanced energy absorption in the active layer is ascribed to the strong local field intensity, surface plasmon polariton (SPP) mode, scattering effect of nanoparticles, and Fabry-Pérot (FP) resonance effect. The SPP mode is bounded to tens of nanometers away from the nanostructure surface and decays exponentially; thus, its effects on the Si film, only 20 nm above the nanostructure, are rather evident. Moreover, the extremely strong intensity of local electricity leads to photonic mode predominant in the active layer and contributes to the energy absorption locally. This mechanism explains better energy absorption at the bottom of the active layer which is just above the nanostructure. The gradient direction of energy absorption (as arrowed in Figure 3f) shows that the energy flux interacts with the back Ag surface at large angles, illustrating that the resonant electromagnetic wave impinges upon the back metal and is reflected into the active layer obliquely. Therefore, the reflection from the back Ag surface, together with the scattering and diffraction caused by the nanostructural geometry, results in strong absorption at the bottom corners of the active layer. The superiority of the lens-like nanoparticle to other structures such as cube, sphere, and cylinder in current enhancement can be attributed to the coexistence of sharp tips and smoothing surfaces - the former helps to induce local surface plasmon and thus the strong field nearby while the latter enables the sunlight to be effectively scattered at large angles. These two factors are significant to solar cells and have been analogically implemented by an array of small spheres distributed on a large sphere [5].

## Conclusions

This work systemically explored the relationship between a variety of complex shapes parameterized by the 3-D Gielis superformula and *J*_sc_. We have found that lens-like nanoparticles can generate significantly higher *J*_sc_ than the two-scale nanospheres that were devised previously. It is important to note that the optimized nanoparticle is more fabrication friendly due to its simpler shape and insensitivity to geometric variations. Numerical simulations have demonstrated that this nanoparticle can yield strong local field enhancement, large scattering angle, Fabry-Pérot resonance, and high surface plasmon polariton, which are suggested as the effective ways to enhance the absorption in the active layer of thin-film solar cells [2].

## Competing interests

The authors declare that they have no competing interests.

## Authors’ contributions

SZ carried out the numerical simulation and optimization. XH participated in the optimization work and carried out the parameter determination and analysis. QL and YX conceived the study and participated in the optimization. All authors participated in the interpretation of the results and revised the manuscript. All authors read and approved the final manuscript.
